# A Prospective Study of Distal Metatarsal Chevron Osteotomies with K-Wire Fixations to Treat Hallux Valgus Deformities

**DOI:** 10.7759/cureus.1704

**Published:** 2017-09-20

**Authors:** MN Baig, Usman Baig, Ali Tariq, Robert Din

**Affiliations:** 1 Orthopaedics, Galway University Hospital; 2 Medicine, Quaid-E-Azam Medical College, Bahawalpur.; 3 Trauma & Orthopaedics, Galway University Hospital; 4 Department of Orthopaedics, Poole Hospital, Poole, United Kingdom

**Keywords:** chevron osteotomy, k wires

## Abstract

Introduction

Hallux valgus is one of the most common forefoot deformities worldwide. Females are affected more often than males. The three most common clinical symptoms are the painful bunion, transfer metatarsalgia, and hammer or claw toes.

Methods

This case series consisted of 20 patients who had chevron osteotomy from January 2015 to January 2016. The clinical assessment was measured by The American Orthopedic Foot and Ankle Score (AOFAS), and radiologic assessment was determined by preoperative and postoperative hallux valgus angle (HVA) and intermetatarsal angle (IMA).

Results

The patients’ mean age was 56 years. Out of 20 patients, 19 were female, and one was male. The mean AOFAS improved from 51 preoperatively to 82 postoperatively. The HVA improved from 26° preoperatively to 14°. There were five complications including four Kirschner (K)-wire complications.

Conclusion

Distal chevron osteotomy is a reliable and time-tested procedure. The K-wire fixation has a relatively high complication rate. We planned to use other methods of fixation and then compared them with K-wires fixation results for future studies.

## Introduction

Hallux valgus is a condition characterized by progressive lateral or valgus deviation deformity of the great toe of the metatarsophalangeal (MTP) joint. There is a prominence at the metatarsal head (bunion) and varus deformity of the first metatarsal. Due to progressive deformity development, it is often accompanied by other foot abnormalities, including secondary osteoarthritis (OA), hammer toes due to crowding, and inflammation of the adventitious bursa over the first MTP joint [1].

The first mention of hallux valgus in the literature was by German surgeon Carl Hueter in 1870 as abd uc  to-valgus deformity of the big toe [2]. Hallux valgus is the most common pathology of the big toe. It is also called as a bunion in the layman terms. This word bunion derives from the Latin word ‘bunio’ meaning turnip. The typical manifestation of the hallux valgus deformity is a prominence of medial eminence. Not all these are hallux valgus deformities, however; it can include bursal inflammation, ganglion formation, osteoarthritis or gouty arthritis, or any combination of these.

In a recent study, the prevalence of hallux valgus was 23% in individuals of 18 to 65 years old, and 35.7% in individuals over the age 65 years. In the juvenile age group, the prevalence is 7.8% [2-3]. The prevalence of hallux valgus by gender was 30% among females and 13% among males [2].

## Materials and methods

**Figure 1:**
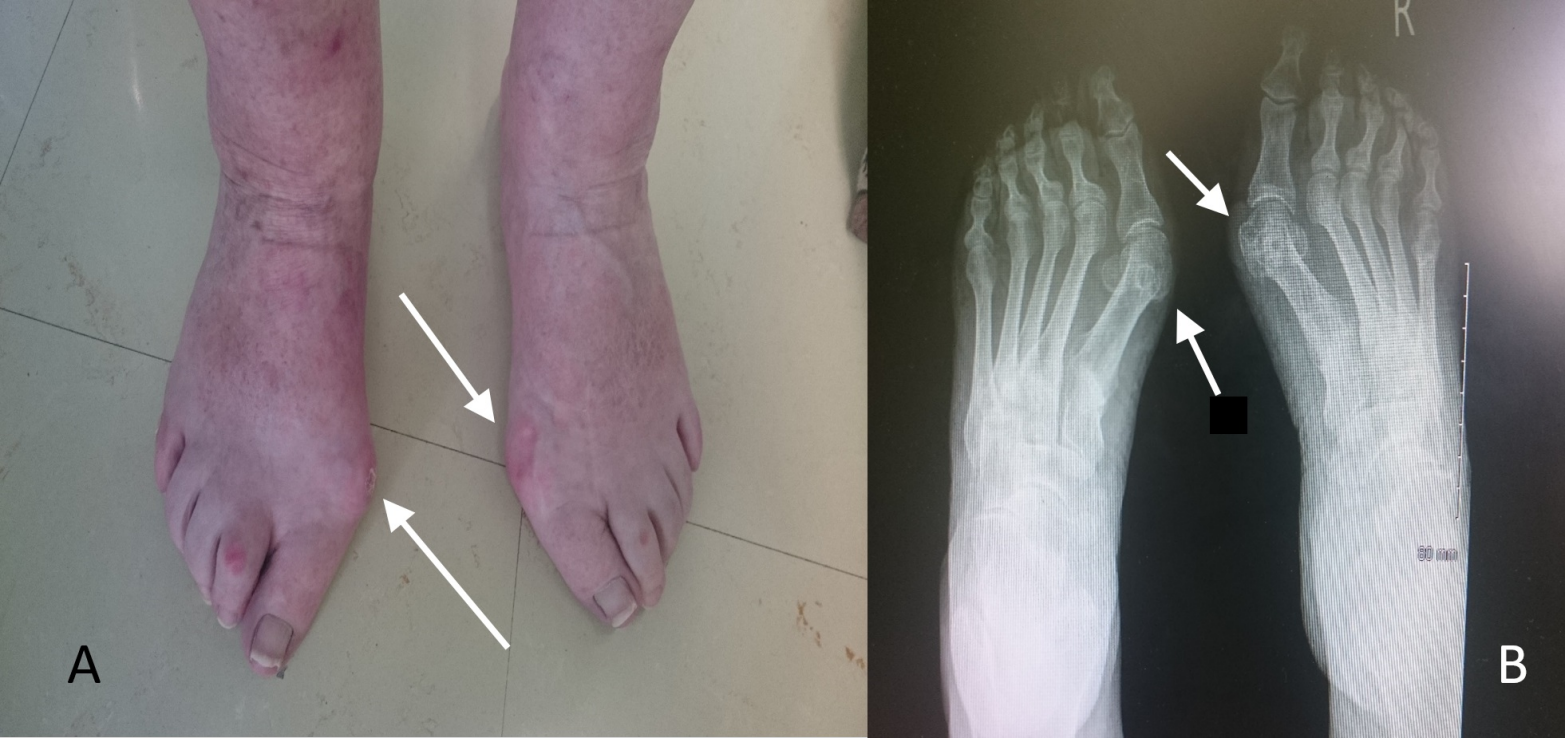
The clinical (A) and radiological (B) presentation of a 62-year-old female with hallux valgus deformity. White arrows showing the clinical and radiological bunion (hallux valgus).

**Figure 2:**
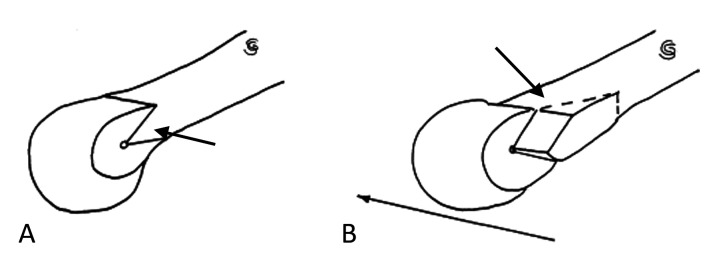
The distal chevron osteotomy “V” cut (A) and translation (B). Black arrows showing the cut and translation.

**Figure 3:**
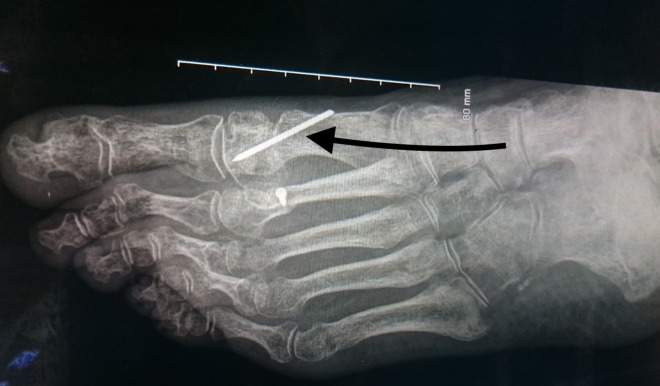
The distal chevron osteotomy and Kirschner (K)-wire fixation. Black arrow showing the Kirschner wire fixation of the distal metatarsal chevron osteotomy.

We performed a prospective study from January 2015 to January 2016. We selected the patients with hallux valgus who underwent distal chevron osteotomy for hallux valgus with Kirschner (K)-wire fixation over that period; the K-wire was chosen as it is cost-effective and technically easier to manipulate. The patients were adults. The surgery was performed by an orthopedic foot and ankle surgeon. There was no randomization or blinding of the patients to this study. The patients’ progress was followed for one year with regular visits as outpatients (Figures 1-3).

The procedure was performed as a day-care surgery. The patients had a preoperative anesthetic assessment performed a few days prior to the surgery. They were assessed by physiotherapists and occupational therapists and briefed on what to expect post operation regarding mobility and rehabilitation.

The data were collected by the orthopedic team via a specific questionnaire made for this study. Postoperatively, the patients were seen in the outpatient department at an interval of two weeks, six weeks, six months and one year. The patients for the study analysis were selected from the outpatient department.

## Results

We used Statistical Package for the Social Sciences (SPSS) Statistics for Windows, Version 19.0 (IBM Corp., Armonk, New York) that was released in the year 2010 for statistically analyzing the data collected from the patients. Wilcoxon signed ranked test was used to evaluate the data. The results obtained from this analysis are shown in Table 1.

**Table 1:** The American Orthopedic Foot and Ankle Score (AOFAS), hallux valgus (HV) angle and intermetatarsal (IM) angle.

n = 20	Preoperative mean (Range)	Postoperative one-year follow-up (Range)	Significance
AOFAS	51 (38 to 60)	82 (75 to 90)	P < .001
HV angle (degrees)	26 (20 to 31)	14 (11 to 18)	P < .001
IM angle (degrees)	13 (11 to 15)	8 (6 to 11 )	P < .001

The patients' age ranged from 28 to 73 years, with a mean age of 56 years. Out of 20 patients who underwent chevron osteotomy and were included in this study, 19 (95%) were female and one (5%) was male. 

The range of the hallux valgus angle (HVA) at the metatarsophalangeal (MTP) joint pre-operatively was 20° to 31°. The postoperative HVA at the one-year follow-up ranged from 11° to 18°. The mean improvement of MTP angle was from 26° to 14° (P < .001).

The range of the intermetatarsal angle (IMA) preoperatively was 11° to 15°. After one year, the range was 6° to 11° (mean=8°). This is a statistically significant improvement (P < .001).

The American Orthopaedic Foot and Ankle Score's (AOFAS) preoperative range was from 38 to 60 (mean=51). At the one-year follow-up, the range was 75 to 90 with a mean of 82 (P < .001).

Postoperatively, complications witnessed included one case of postoperative pain and four K-wire complications. The patient who experienced pain was a female who reported pain at the sixth-month and one-year visit. She is being treated symptomatically with topical analgesic and oral analgesic. Four patients had K-wire complications including pin site infection and K-wire migration. Two patients had a K-wire migration of approximately 1 cm to 1.5 cm during the intervals of a two-week visit. The other two patients had superficial pin site infections; these were treated with antibiotics for five days.

## Discussion

There are many different types of osteotomies for the correction of the hallux valgus deformity. There are different osteotomies according to anatomical sites. Distal chevron osteotomy has been considered one of the most successful types of osteotomy as it has demonstrated good results in the medical literature. Though it has better results in comparison to the McBride technique and Wilson osteotomy [4-5], when compared to the Scarf osteotomy in randomized trials by Jeuken, et al. and Deenik, et al., the results were similar in both groups [6-7]. The technique of ‘V’ shaped osteotomy and lateral displacement is considered as a big step in the correction of hallux valgus and has been proven effective [8-9]. Table 2 shows different types of osteotomies.

**Table 2:** Different hallux valgus correction osteotomies according to the site of correction.

Metatarsal	Metatarsophalangeal joint	Phalanx
Basal osteotomy, Chevron osteotomy, Hohmann osteotomy, Hueter osteotomy, Kramer osteotomy, Scarf osteotomy, Ludloff osteotomy.	Metatarsophalangeal (MTP) joint arthrodesis, McBride soft tissue balancing procedure.	Akin osteotomy, Keller-Brandes osteotomy.

In this study, the mean improvement in the HVA was from 26° to 14° (P < .001). According to the available literature, Chevron osteotomy has been shown to deliver significant improvement in the HVA. Schneider, et al. showed an improvement in the HVA from the mean of 27.6° to 14.0° [9]. Likewise, IMA has also shown a statistically significant improvement from a mean value of 12.90° preoperatively to 8.45° postoperatively. The AOFAS significantly improved from 50.70 to 82.4. The literature has repeatedly shown improvements in the HVA and IMA after chevron osteotomy [10-12]. The chevron ‘V’ osteotomy is a biplane osteotomy with the transverse plane and vertical plane. It is inherently stable in only one plane (vertical plane) and is not stable in the transverse plane. The choice of fixation after an osteotomy is dependent on the surgeon’s preference, patient compliance, bone stock, and osteotomy type [12].

Different methods of fixation are used, including K-wire, Herbert-Whipple screw, suture anchors or bio-absorbable fixations like polyglycolide pins, staple fixation and plate fixation [9,11]. All these methods of fixation have been used and their results are discussed in the literature; this discussion is confined to our experience with K-wire fixation. It is a popular method because it is a cheap and simple method [12-13]. It is also very easy to remove during the outpatient visits. We used a 1.6-mm K-wire across the osteotomy sites. The K-wires in our study were taken out during outpatient visits at six weeks.

The complications which are generally attributed to K-wire fixation include pin site infection, proximal displacement, distal displacement, pain, lack of interfragmentary compression, no resistance to parallel movement of the wire, and heat generated during insertion [14-15]. Additionally, K-wires can be prone to infection because one end of the K-wire is out of the skin. In comparison with other methods, some studies from the 1990s and early 2000s demonstrated either positive results and relative benefits with K-wire fixation or that the method was at least of equal efficacy [15-16]. In contrast, there have been a number of studies in the literature illustrating superior results for other methods in comparison to K-wire fixation. In a study by Acevedo, et al., it was shown that regardless of the type of osteotomy, screw fixation was mechanically superior to other methods including K-wire fixation [15].

## Conclusions

This study showed that a distal chevron osteotomy can be used successfully for the correction of hallux valgus deformity. Analysis of the preoperative and postoperative values for the HVA, IMA, and AOFAS show statistically significant improvement in all categories measured, both radiologically and clinically. In line with our clinical experience, the rate of K-wire complications in this study was quite high (20%). Many of the complications such as displacement and pin site infection are preventable. The limitation of this study was the small number of participants. To improve our practice and reduce the complication rate, our aim would be to use other methods of fixation instead of k-wire fixation and study their complication and problems to come up with the result of the most appropriate method of fixation.
